# Renal-Limited Amyloid Light-Chain (Lambda) Amyloidosis Presenting as Monoclonal Gammopathy of Renal Significance: A Case Report

**DOI:** 10.7759/cureus.105750

**Published:** 2026-03-24

**Authors:** Sritharan Thivacaren, Anura P Hewageegana, Priyani Amarathunga, Mihiran Thanigasalan

**Affiliations:** 1 Department of Nephrology, National Hospital of Sri Lanka, Colombo, LKA; 2 Department of Pathology, Faculty of Medicine, University of Colombo, Colombo, LKA

**Keywords:** congo red, mass spectrometry, monoclonal gammopathy of renal significance (mgrs), nephrotic syndrome, renal al amyloidosis

## Abstract

Monoclonal gammopathy of renal significance (MGRS) encompasses renal diseases caused by nephrotoxic monoclonal immunoglobulins produced by small B-cell or plasma cell clones that do not meet the criteria for overt hematologic malignancy. Renal amyloid light-chain (AL) amyloidosis is a recognized lesion within the MGRS spectrum. We report the case of a 66-year-old woman who presented with nephrotic syndrome and renal dysfunction associated with monoclonal lambda light chains. Renal biopsy confirmed AL (lambda) amyloidosis, while bone marrow examination and systemic evaluation revealed no evidence of multiple myeloma or systemic amyloid deposition. This case highlights that early recognition of MGRS-associated renal amyloidosis is essential, as delayed therapy may contribute to preventable morbidity and mortality.

## Introduction

Amyloidosis is a heterogeneous group of disorders characterized by extracellular deposition of insoluble fibrillar proteins arranged in a cross-β-pleated sheet structure [1]. More than 30 amyloidogenic precursor proteins have been identified. Classification is based on the biochemical composition of the fibril protein [2]. Systemic amyloidosis is broadly categorized into amyloid light-chain (AL) amyloidosis, serum amyloid A (AA) amyloidosis, hereditary amyloidosis (most commonly transthyretin-related), and, less frequently, leukocyte chemotactic factor 2 (ALECT2) and β2-microglobulin amyloidosis [2]. AL amyloidosis is the most common form of systemic amyloidosis, with an estimated incidence of approximately 8-12 cases per million person-years [3]. Renal-limited AL amyloidosis is very uncommon, occurring in fewer than 5-10% of AL amyloidosis cases at presentation (fewer than one case per million/year) [4]. AL amyloidosis is frequently associated with plasma cell dyscrasias; however, some patients lack myeloma-defining features and are classified as having monoclonal gammopathy of renal significance (MGRS). Renal AL amyloidosis is a recognized lesion within the MGRS spectrum and typically presents with nephrotic-range proteinuria. The kidney is the most commonly involved organ in AL amyloidosis, with renal involvement reported in up to 70% of cases. This case highlights renal AL amyloidosis presenting within the MGRS spectrum, confirmed on kidney biopsy despite the absence of myeloma-defining features or demonstrable systemic amyloid involvement. Early diagnosis is essential as renal outcomes correlate strongly with the degree of proteinuria and baseline renal function at presentation [5]. Delayed recognition may result in irreversible kidney damage and progression to end-stage kidney disease.

## Case presentation

A 66-year-old woman with a background history of hypertension and dyslipidemia presented with generalized body swelling, frothy urine, and progressive fatigue for one month. She denied hematuria, dysuria, oliguria, weight loss, anorexia, fever, back pain, or other systemic symptoms suggestive of malignancy. She had been diagnosed with hypertension 10 years earlier and was compliant with regular antihypertensive therapy. There was no history of native or herbal medication use, long-term analgesic intake, or known autoimmune disease.

On examination, she had bilateral pitting pedal edema extending up to the knees with clinical evidence of ascites. She appeared pale, with no lymphadenopathy or hepatosplenomegaly. Her blood pressure was 140/90 mmHg, and pulse rate was 76 beats per minute and regular. Cardiovascular examination revealed no features of heart failure, and neurological examination was normal.

**Figure 1:**
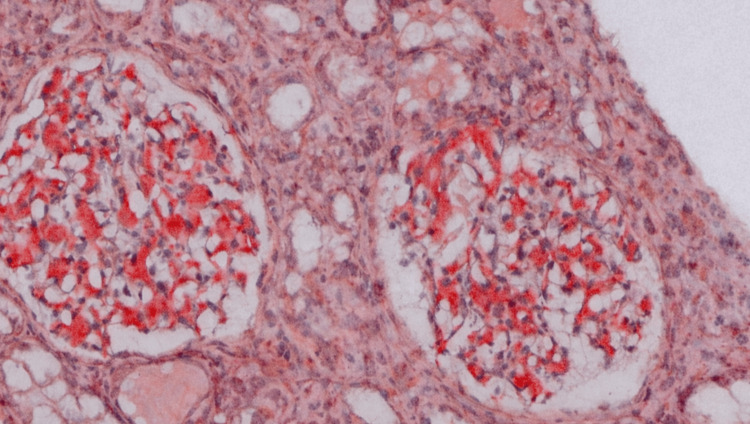
Congo red staining demonstrating amyloid deposition (×200).

**Figure 2:**
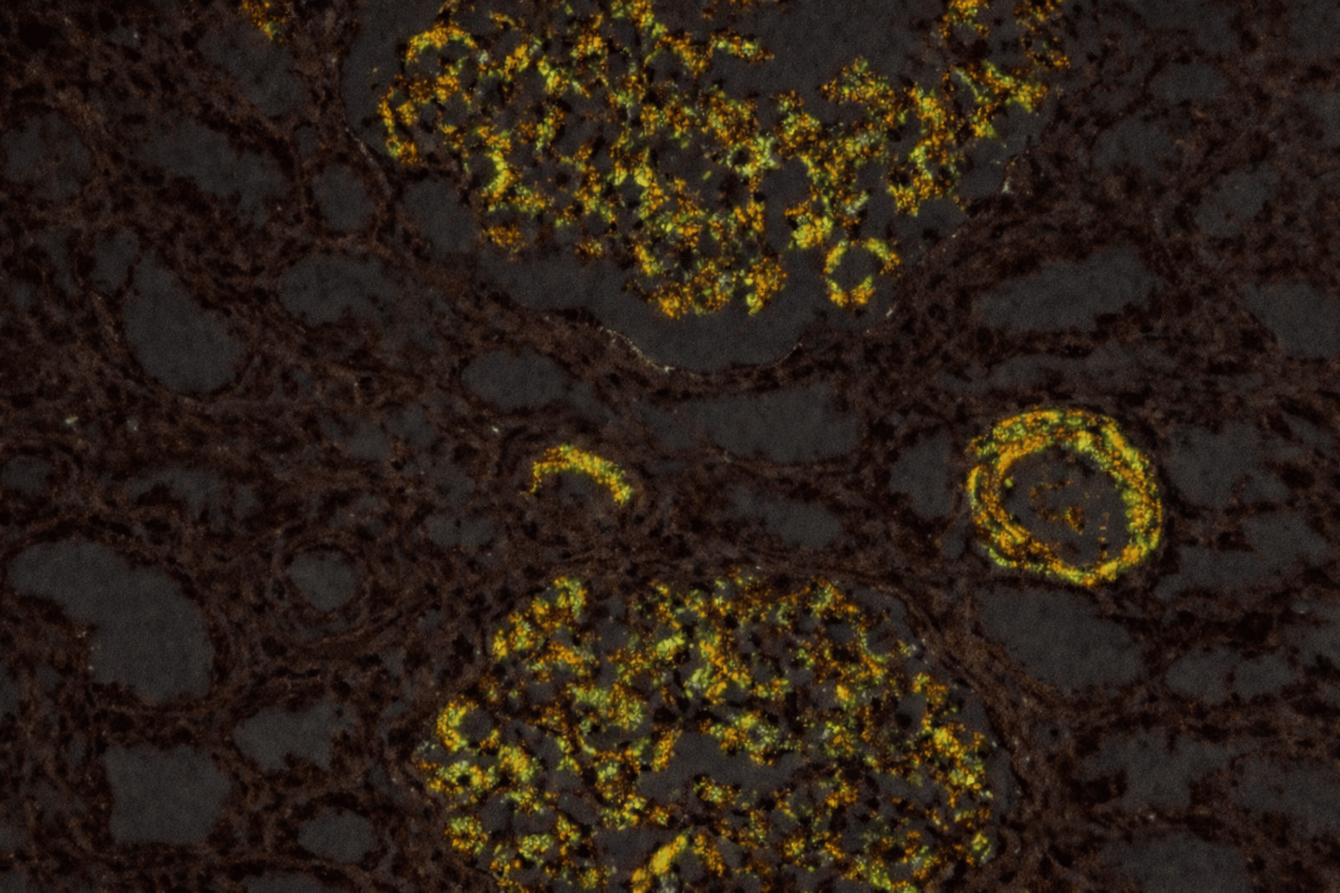
Apple-green birefringence under polarized light confirming amyloid (×200).

Baseline laboratory investigations are summarized in Table 1. Twenty-four-hour urinary protein excretion was markedly elevated at 12,275 mg/day. Blood picture revealed mild anemia and moderate rouleaux formation. Serum protein electrophoresis demonstrated a monoclonal band in the gamma region. Serum free light-chain assay revealed markedly elevated lambda light chains (178 mg/L) with an abnormal kappa-to-lambda ratio. Renal biopsy showed extensive mesangial deposition of amyloid, confirmed by Congo red positivity (Figure 1) with apple-green birefringence under polarized light microscopy (Figure 2). Immunofluorescence demonstrated strong (3+) granular staining for lambda light chains. Bone marrow biopsy did not reveal significant plasmacytosis. Congo red staining in bone marrow was negative. Clonal characterization was limited due to a lack of advanced hematologic studies. Repeated abdominal fat pad biopsies were negative. Her chest X-ray was normal. Contrast-enhanced CT of the chest, abdomen, and pelvis revealed moderate ascites with no definitive masses or features suggestive of chronic liver cell disease. Skeletal survey revealed no evidence of lytic lesions. Two-dimensional echocardiogram revealed an ejection fraction of 80% with good left ventricular function and no evidence of restrictive filling pattern or diastolic dysfunction.

**Table 1:** Key laboratory investigation findings. WBC = white blood cell; TSH = thyroid-stimulating hormone; ANA = antinuclear antibody; HbsAg = hepatitis B surface antigen; HbcAb = hepatitis C antibody; CA125 = cancer antigen 125; HbA1c = hemoglobin A1c

Parameter	Value	Reference range
Full blood count		
WBC (10^9^/L)	8.7	4.0–10.0
Neutrophils (10^9^/L)	6.0	2.0–7.0
Lymphocytes (10^9^/L)	2.7	0.8–4.0
Hemoglobin (g/dL)	7.9	11–16.0
Platelets (10^9^/L)	414	150–450
Erythrocyte sedimentation rate (mm/hour)	48	0–20
Serum sodium (mmol/L)	133	136–145
Serum potassium (mmol/L)	4.1	3.5–5.1
Corrected calcium (mg/dL)	9.9	8.5–10.5
Serum creatinine (mg/dL)	2.24	0.6–1.1
Aspartate aminotransferase (U/L)	26	5.0–34
Alanine aminotransferase (U/L)	13	0–55
Urine full report		
Albumin	2+	Nil
Pus cells (/hpf)	1–2	0–5
Red cells (/hpf)	Nil	0–2
24-hour urinary protein (mg/24 hours)	12,275	150 mg/24 hours
Urine protein creatinine ratio (mg/mg)	23.99	Less than 0.15
Total protein (g/dL)	4.0	6.4–8.3
Albumin (g/dL)	1.1	3.5–5.2
Globulin (g/dL)	2.9	2.2–4.0
Serum free light-chain assay (mg/L)	Kappa light chains = 18.3	Kappa = 3.3–19.4
	Lambda light chains = 178	Lambda = 5.7–26.3
HbA1c (%)	5.2%	Less than 5.7%
TSH (mIU/L)	3.8	0.4–4.0
Immunological markers		
ANA	Negative	Negative
HbsAg	Negative	Negative
HbcAb	Negative	Negative
HIV I and 2	Negative	Negative
C3 (mg/dL)	100	90–180
C4 (mg/dL)	20	10–40
CA125 (U/mL)	132	Less than 35
Alpha-fetoprotein (ng/mL)	10.0	Less than 10 ng/mL
Two-dimensional echocardiography	Ejection fraction of 70%, with good left ventricular systolic function. No features of restrictive pattern or diastolic dysfunction. A normal E/A ratio	Normal
Troponin (ng/mL)	Negative	Less than 0.04
N-terminal pro-B-type natriuretic peptide (pg/mL)	600	Less than 700

Renal amyloidosis was diagnosed following extensive clinical, laboratory, and histopathological evaluation. After multidisciplinary discussion, she was planned for initiation of a bortezomib, thalidomide, and dexamethasone-based chemotherapy regimen under the care of the hematology team. Warfarin at a dose of 5 mg daily was commenced as thromboprophylaxis. However, before the initiation of definitive therapy, the patient developed severe pneumonia complicated by septic shock. Despite appropriate supportive management, her clinical condition deteriorated, and she succumbed to the illness.

## Discussion

This patient fulfilled the diagnostic criteria for MGRS, defined by renal injury caused by a monoclonal immunoglobulin in the absence of myeloma-defining events or overt lymphoproliferative malignancy. MGRS encompasses renal disorders caused by nephrotoxic monoclonal immunoglobulins produced by small B-cell or plasma cell clones that do not meet the criteria for overt hematologic malignancy [6]. Early recognition is critical, as even low-level monoclonal protein production can result in progressive and irreversible kidney damage [6]. Renal AL amyloidosis is a recognized pathological entity within the MGRS spectrum [7]. It typically presents with nephrotic-range proteinuria and renal dysfunction [8]. AL amyloidosis should be considered in patients with nephrotic syndrome and a monoclonal band or abnormal serum free light-chain ratio, even if there are no systemic symptoms.

Although AL amyloidosis is most commonly associated with multiple myeloma, some patients lack myeloma-defining features or systemic organ involvement. In such cases, renal disease may represent the sole manifestation of monoclonal immunoglobulin deposition, fulfilling the diagnostic criteria for MGRS. Our patient exhibited nephrotic syndrome, renal impairment, and monoclonal lambda light-chain excess and no clinical, biochemical, cardiac (echocardiography), or radiological or histological evidence of extrarenal amyloid involvement supporting the diagnosis of renal-limited AL amyloidosis in contrast to typical systemic AL amyloidosis, which most commonly involves the kidneys (70%), heart (50-60%), as well as the liver and peripheral nervous system. Renal-limited AL amyloidosis is characterized by the absence of cardiac involvement, reflected by normal cardiac biomarkers and unremarkable echocardiographic findings. In contrast, systemic AL amyloidosis frequently involves the heart, leading to elevated cardiac biomarkers (e.g., N-terminal pro-B-type natriuretic peptide) and typical echocardiographic features, such as increased ventricular wall thickness and diastolic dysfunction [9]. Renal-limited AL amyloidosis is associated with a significantly better prognosis than systemic AL amyloidosis, primarily due to the absence of cardiac involvement [9].

Renal biopsy is indispensable for diagnosing MGRS-related kidney disease. Congo red positivity with apple-green birefringence and light-chain restriction on immunofluorescence establishes the diagnosis and prevents delay. Differential diagnoses of nephrotic syndrome with monoclonal gammopathy include light-chain deposition disease and fibrillary glomerulonephritis, which were excluded by Congo red positivity and immunofluorescence findings [10]. The markedly elevated serum free lambda light chain with an abnormal kappa-to-lambda ratio supported a pathogenic monoclonal process despite the absence of myeloma-defining features.

Management of MGRS focuses on suppressing the pathogenic monoclonal clone, irrespective of tumor burden. Absence of myeloma-defining features does not exclude pathogenic monoclonal disease. Renal AL amyloidosis can present as MGRS and still warrants clone-directed evaluation and therapy planning. Bortezomib-based regimens are first-line therapy in AL amyloidosis due to their rapid reduction of free light-chain levels. Patients with nephrotic syndrome and AL amyloidosis are at high risk for early complications (infection and thrombosis). Hence, management should be multidisciplinary with careful supportive care alongside planned chemotherapy. Mass spectrometry should be considered the gold standard for typing amyloid fibrils in routine practice [11]. Unfortunately, Sri Lanka does not have mass spectrometry-based amyloid typing, and there is a lack of longitudinal follow-up of amyloid patients due to early mortality. Although renal-limited AL amyloidosis generally carries a more favorable prognosis, our patient succumbed before the initiation of definitive therapy. This adverse outcome was likely multifactorial, reflecting advanced age, frailty, multiple comorbidities, delayed referral from a peripheral healthcare setting, and limited access to advanced diagnostic modalities such as cardiac MRI, mass spectrometry, and electron microscopy.

## Conclusions

Renal-limited AL amyloidosis is an uncommon manifestation of MGRS and may present without the classical features of overt plasma cell dyscrasia or multiple myeloma. This case highlights the importance of maintaining a high index of suspicion in patients presenting with nephrotic syndrome and evidence of monoclonal gammopathy, even in the absence of myeloma-defining events. Renal biopsy remains the cornerstone of diagnosis, as it enables definitive identification of amyloid deposition and accurate typing via Congo red staining and immunofluorescence. This case underscores the need for a multidisciplinary approach involving nephrologists, hematologists, and pathologists to ensure accurate diagnosis and optimal management. Increased awareness of diagnosing renal-limited AL amyloidosis as a form of MGRS will facilitate appropriate treatment, ultimately improving renal and overall patient outcomes.
